# Evaluation of Antibiotic Use in Kazakhstan for the Period 2017–2019 Based on WHO Access, Watch and Reserve Classification (AWaRe 2019)

**DOI:** 10.3390/antibiotics10010058

**Published:** 2021-01-08

**Authors:** Gulzira Zhussupova, Dinara Utepova, Galiya Orazova, Saule Zhaldybayeva, Galina Skvirskaya, Kanat Tossekbayev

**Affiliations:** 1 The Republican State Enterprise on the Right of Economic Management “Republican Center for Health Development”, the Ministry of Health of the Republic of Kazakhstan, 010000 Nur-Sultan, Kazakhstan; utepova-88@mail.ru (D.U.); zhaldybaeva71@mail.ru (S.Z.); tossekbayew_kana@mail.ru (K.T.); 2 Department of Public Health, Astana Medical University, 010000 Nur-Sultan, Kazakhstan; 3 N.A. Semashko Department, Public Health and Healthcare I.M. Sechenov First Moscow State Medical University (Sechenov University), 119991 Moscow, Russia; gskvirskaya@mail.ru

**Keywords:** antimicrobial medicines, antibiotic consumption, AWaRe, Essential Medicines List, Kazakhstan

## Abstract

The purpose of this study is to conduct a comparative analysis of the consumption of antibiotics for systemic use reimbursed by the state in Kazakhstan for 2017–2019 with the Access, Watch, and Reserve classification (AWaRe 2019) of the World Health Organization (WHO). The evaluation of the consumption of antibiotics for systemic use in Kazakhstan for 2017–2019 was carried out using the ATC/DDD methodology in accordance with the WHO AWaRe classification. The study used data on all antibiotics that were centrally purchased by a single purchaser during the study period. To understand how often Access group antibiotics are taken in Kazakhstan, the top-10 most consumed antibiotics were additionally studied. The results of a comparative analysis of the antibiotics for systemic use consumption for 2017–2019 by the Access, Watch, and Reserve groups showed a negative trend of a decrease in the consumption of Access group drugs from 1.17 defined daily dose (DDDs) per 1000 inhabitants per day (DID) (39%) in 2017 to 0.59 DID (30%) in 2019. There is an increase in consumption of Watch group antibiotics from 1.84 DID (61%) in 2017 to 1.37 DID (68%) in 2019, as well as an increase in consumption of Reserve antibiotics from 0.001 DID (0.03%) to 0.4 DID (2.11%). In recent years in Kazakhstan, there has been a decrease in the consumption of Access group antibiotics. In addition, the Watch group antibiotics are widely consumed with a certain upward trend. In 2019, one Reserve antibiotic was included in the top-10 most commonly consumed antibiotics. There is a predominant consumption of parenteral forms of antibiotics for systemic use in the country.

## 1. Introduction

Antimicrobial resistance (AMR) is a global problem of public healthcare that comes with huge social and economic losses due to infectious diseases. The potential for widespread use of antimicrobials in medical practice could lead to long-term increases in AMR mortality rates.

To optimize the rational use of antibiotics and support monitoring, in March 2017, the WHO introduced a detailed classification of antibiotics, which was designated Access, Watch, and Reserve [1,2]. This classification includes information about 180 antibiotics classified as Access, Watch, and Reserve and also pharmacological classes, anatomical—therapeutic classification codes, and status in Essential Medicines List (EML) WHO. The AWaRe classification also lists the antibiotics that are not recommended by WHO, especially combinations with fixed doses of several broad-spectrum antibiotics that do not have proven indications or recommendations in high-quality international guidelines [3]. In 2019, the above-mentioned classification was updated [4]. WHO’s 13th General Programme of Work (GPW 13) requires at least 60% of Access antibiotics to be consumed to achieve health-related sustainable development targets [2].

Kazakhstan, as part of the global community, adheres to the WHO recommendations. However, despite a slight decrease in the consumption of antimicrobials for systemic use in recent years, there is still an irrational use of antibiotics in Kazakhstan. This is due to their nonprescription (27.5% of antibiotics are taken without a doctor’s prescription) and excessive prescription (the share of antibiotic prescriptions—29.9% of all drugs, which exceeds the WHO recommended level—20%) leads to an increase in AMR [5]. The lack of a national strategy for containment of AMR reduces the effectiveness of the prevention of infectious diseases (for example, despite the efforts to combat tuberculosis, Kazakhstan occupies 92nd place among 138 countries on the factor of the prevalence of the disease [5,6]). In addition, we are concerned that this trend will intensify as a result of the widespread empirical use of antibiotics during the COVID-19 pandemic.

Until now, in Kazakhstan, single pharmacoepidemiological studies have been carried out to determine the effectiveness and safety of the use of drugs at the population level. Even though in recent years, extensive activities have been carried out to raise awareness of the population and medical workers, the problem of irrational consumption of antibiotics is still a complex issue of national health care. The main volume of medical care provided to the population of Kazakhstan is reimbursed by either the state or the social health insurance fund. Therefore, the study of the consumption of antimicrobial drugs in the country’s public medical institutions will make it possible to assess the real picture and develop effective measures to contain antimicrobial resistance.

### Aim of Study

This study aims to conduct a comparative analysis of the consumption of antimicrobials for systemic use reimbursed by the state in Kazakhstan for 2017–2019 with the WHO AWaRe classification.

## 2. Results

The results of the evaluation of antibiotic consumption for systemic use showed a decrease in total antibiotic consumption from 3.03 DID in 2017 to 2.66 DID in 2018 and 2.00 DID in 2019. Along with this, there was a decrease in the proportion of consumption of oral forms of antibiotics from 48.6% and 46.8% in 2017 and 2018 to an average of 9.2% in 2019 (38.5%), and when comparing 2017 and 2019, a decrease to 10.1%.

The antibiotics of the group “J01D Other Beta-lactam antibacterials” lead in terms of consumption share in 2019, accounting for 50% (1.01 DID) of total consumption, followed in descending order, groups “J01M Quinolone antibacterials”, “J01F Macrolides, Lincosamides, and Streptogramins” and “J01G Aminoglycosides”, with a 30% (0.59 DID), 10% (0.2) and 7% (0.15) consumption share respectively.

More detailed analysis of the “J01D Other beta-lactam antibacterials” preparation group showed that cephalosporins generations I and III took a leading position in terms of consumption in 2019, which was 43.6% and 45.6%, respectively, and 8.5% and 0.8% for generations II and IV, followed by carbapenems (Meropenem, Ertapenem, Doripenem, Imipenem / Cilastatin—in descending order). Levofloxacin takes the first place among the drugs of the J01M Quinolone antibacterials group, the share of consumption of which is 74.1%.

An analysis of the consumption dynamics of group “J01C Beta-lactam antibiotics, penicillins” revealed a significant increase in consumption of subgroup “J01CR02 Amoxicillin and beta-lactamase inhibitor” in 2019, while the drug Amoxicillin was not procured.

One hundred and seventy-two positions of the 180 antimicrobial drugs according to the WHO AWaRe-2019 antibiotic classification are from the group of antibiotics for systemic use J01; 8 of these positions had other ATC codes: A07AA09, A07AA11, P01AB01 and 5 were “to be assigned”. This includes a total of 75 antibiotics in the EML WHO for 2019, of which 71 are for systemic use. In Kazakhstan, during the period from 2017 to 2019, only 35 state reimbursable antibiotics for systemic use reimbursed by the state were consumed.

In 2017 and 2018, the pattern of antibiotic consumption did not change. The list of the Single Purchaser of Kazakhstan included 34 antibiotics for systemic use J01, including 21 items from the WHO EML list. Thirty-three out of 34 antibiotics are included in the WHO AWaRe classification of antibiotics (11—Access, 21—Watch; 1—Reserve), and 1 drug (Cefoperazone and Sulbactam) is included in the WHO non-recommended list.

In 2019, the number of consumed antibiotics decreased to 29; 17 of which are included in the WHO EML. Among the 29 preparations, six were Access, 21 were Watch, and two were from the Reserve group (Fosfomycin (IV)) and Linezolid) (Table 1).

Twenty-two antibiotics out of all 34 included in the WHO AWaRe classification are included in the WHO EML (10—Access, 10—Watch, 2—Reserve), and 12 are not included in the WHO EML (1—Access, 11—Watch). The comparison results are presented in Table 2 and Table 3.

**Figure 1:**
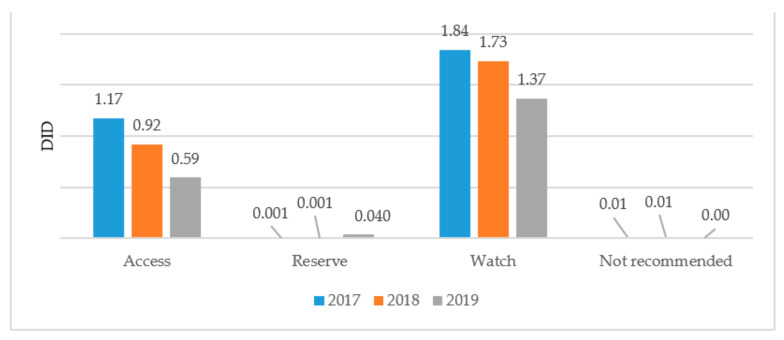
Results of antibiotic consumption in Kazakhstan by AWaRe classification for 2017–2019.

A comparative analysis of antibiotic consumption for systemic use in 2017–2019 by Access, Watch, and Reserve groups shows a negative trend of decreasing consumption of drugs from the Access group from 1.17 DID (39%) in 2017 to 0.59 DID (30%) in 2019. There is an increase in consumption of Watch group antibiotics from 1.84 DID (61%) in 2017 to 1.37 DID (68%) in 2019, as well as an increase in the consumption of Reserve antibiotics from 0.001 DID (0.03%) to 0.4 DID (2.11%). At the same time, in 2017 and 2018, in the list of a Single Purchaser, there was one combined antibiotic included in the WHO list of antibiotics that is not recommended for consumption (Figure 1).

**Figure 2:**
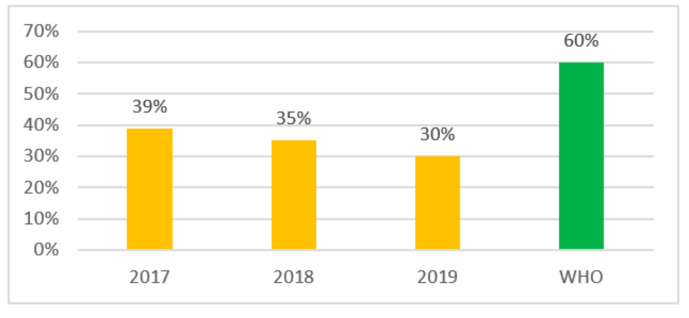
Indicator of consumption of Access antibiotics in Kazakhstan.

Going forward we checked the compliance with the WHO recommendations for the indicator level of the use of the Access group antibiotics—at least 60% for 3 years (2017–2019) and received the following results: in 2017 the indicator level was 39%, and it was 35% in 2018 and 30% in 2019 (Figure 2). Therefore, in Kazakhstan there is a reduction in consumption of Access group antibiotics from 39% to 30% during the period 2017–2019 (Figure 2).

**Figure 3:**
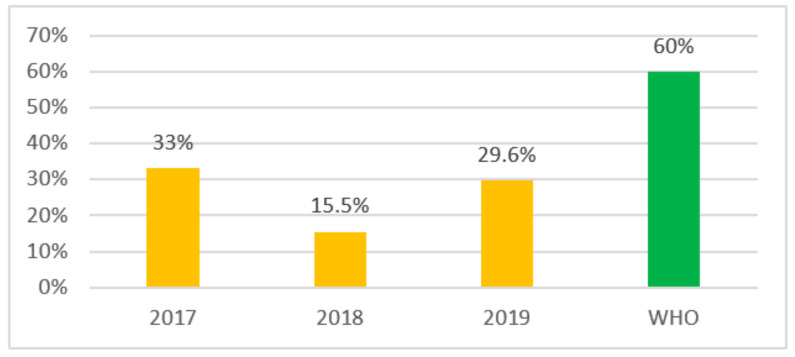
Access group antibiotics use indicator by the most used top 10.

In order to understand how often Access antibiotics are used in Kazakhstan we looked at the top 10 of the most consumed antibiotics. The results of determining the indicator of antibiotic consumption at the country level according to the top 10 showed a decrease in the share of use of Access group antibiotics by almost 2 times in 2018, which amounted to 15.5% compared to 2017—33%, and there was a reverse increase in 2019 to 29.6%. The obtained results are presented in Figure 3.

At the same time, most of the top 10 parenteral and oral antibiotics in 2019 were Watch group drugs with a share of the total consumption of 66.1%, which is 13.1% less than 2018 (79.2%) and 4.5% more than 2017 (61.6%) (Table 4, Table 5 and Table 6).

## 3. Discussion

In general, over the period under review, we identified a negative trend in the consumption of systemic antibiotics in Kazakhstan in all three categories of the WHO AWaRe classification. Despite a positive systematic decrease in the total consumption of antibiotics from 3.03 DID in 2017 to 2.66 DID in 2018 and 2.00 DID in 2019, there is a negative decrease in the share of consumption of oral forms of antibiotics by 10.1% in 2019, which amounted to 38.5% of the total consumption compared to 2017 (48.6%).

In addition, in 2019, there is a stable leading position of antibiotics of the group “J01D Other beta-lactam antibacterial drugs”, with a consumption share of 50% (1.01 DID) of total consumption, among which the most consumed were cephalosporins of the third generation with a consumption share within the group of 45.6%. At the same time, according to the WHO AWaRe classification, 94% (17 out of 18) of generation III cephalosporins belong to the Watch group, which indicates the irrationality of the structure of consumption of antibiotics for systemic use.

Analysis of overall antibiotic consumption compliance with WHO guidelines over the study period showed a systematic decrease in the consumption of Access antibiotics from 39% in 2017 to 35% in 2018, reaching 30% in 2019.

More than half of the prescribed antibiotics in Kazakhstan were from the Watch group. Over the period studied, we found such a negative trend that the share of consumed Watch group antibiotics increased from 61.6% (2017) to 66.1% (2019). For comparison, in one of England’s clinics, the share of consumption of Access group antibiotics was 60.9% in 2016, and Watch was 37.9% [7].

Meanwhile, the use of Access group antibiotics among the most commonly used top 10 antibiotics varied over the study period and declined sharply from 33% (2017) to 15.5% in 2018 and increased again to 29.6% in 2019. However, the WHO recommended indicator level of at least 60% use of Access antibiotics was not achieved [2].

In the available literature, there were interesting results of a comparative analysis of data from 70 countries of the world with middle and high income on the use of oral forms of antibiotics in children based on WHO AWaRe in 2015 [8]. According to the authors, the average consumption of antibiotics in this category is 76%. In only 17 (24%) out of 70 countries, the share of Access antibiotics use was less than 63%. According to the same authors, Kazakhstan takes 15th place in the prescription of Access group antibiotics for the child population (85%). Incidentally, the highest rate was found in Slovenia (94.4%), and the lowest in China (27.0%). Analyzing these data on the use of systemic antibiotics in children, we believe that, in Kazakhstan, there is irrational use of antibiotics with a predominance of consumption of Watch group antibiotics at the expense of the adult population. We believe that this is most likely due to the presence of resistance to Access antibiotics in the adult category of patients.

In 2019, Linezolid, which belongs to the Reserve antibiotics group according to the WHO AWaRe classification, entered the top 10 most consumed oral antibacterial drugs, with a consumption share of 2.1%, which poses a threat to the development of resistance to reserve drugs that should be prescribed under strict control and reporting on the reasonableness of the prescription. For comparison, in Italy, the daily consumption of “reserve” antibiotics is 2.11% of the total. This is four times the number in Germany and six times the rate in the UK, where the daily antibiotic consumption of last resort is 0.3% [9].

If the same antibiotic has both forms (oral and parenteral) and the possibility of use, the most appropriate is the use of oral forms of antibiotics [10]. The predominant use of parenteral antibiotics will inevitably lead to an increase in injection complications, an increase in costs due to the higher cost of parenteral antibacterial drugs, as well as an increase in the workload of hospital staff. To assess the consumption of systemic antibiotics by route of administration, we compared the data obtained with the results of our previous studies [11]. The analysis showed a negative trend towards an increase in the use of parenteral forms of antibiotics. Therefore, in 2019, the share of their consumption increased by 10.1%, amounting to 61.5% of the total consumption of antimicrobial drugs in 2019 compared to 2017 (51.4%). At the same time, total consumption decreased by 16% and amounted to 1.23 DID in 2019 compared to the figure for 2017 (1.46 DID). This is alarming given that, until 2017, drugs had been centrally purchased only for the hospital level. From 2018, a single purchaser began to additionally procure drugs for the outpatient level.

According to WHO estimation, there are seven factors promoting irrational antibiotics use:

(1) lack of skills and knowledge of the prescriber;
(2) improper unethical promotion of medicines by pharmaceutical companies;
(3) unlimited availability of medicines;
(4) profit from the sale of drugs;
(5) drugs that are not affordable;
(6) excessive workload of medical personnel;
(7) lack of a coordinated national pharmaceutical policy [12].

Based on this, we believe that one of the main reasons for the inappropriate use of antibiotics in Kazakhstan is the lack of guidance on antibiotic use at the national level and a lack of human resources. In total, 59% of medical institutions in the country do not have clinical pharmacologists responsible for achieving the effectiveness and safety of pharmacotherapy [5]. Along with this, in most developed and some developing countries (Canada, USA, Kingdom of Saudi Arabia, Australia, China, Egypt, India, etc.), the functions of clinical pharmacologists for the rational use of medicines are successfully performed by clinical pharmacists, whose main responsibilities are to check prescriptions and advise medical staff and patients on the use of drugs [13,14,15,16,17,18,19]. Clinical pharmacists and general pharmacists can detect and correct errors in prescriptions by reviewing medical prescriptions and reduce costs associated with misuse, errors and avoidable adverse events [20,21]. In addition, the implementation at the local level of the practice of analyzing the consumption of antibiotics will allow a comparison of the absolute consumption of antibiotics in the context of a particular medical organization for previous years, and then, using data on absolute consumption, make a relative comparison in accordance with the AWaRe categories.

For the rational use of antibiotics in Kazakhstan and optimization of procurement, there is a need to decentralize the procurement of antimicrobial drugs. This practice has long been widespread in many countries of the world. Although there is evidence on the negative aspects of the policy of decentralizing the procurement of antibiotics in the available literature, such as problems of logistics and regulation [22], the independent procurement of antibiotics by medical organizations will allow local sensitivities to certain drugs to be taken into account [23]. We believe that decentralizing the procurement of antimicrobial drugs could be a major step in controlling AMR in public health facilities in Kazakhstan.

Taking into account the results of the study, where the consumption of the Access group was 29.6% (<60% according to WHO), as well as the irrational use of antibiotics (excessive consumption of Watch, and Reserve antibiotics) in the country, it is necessary to introduce the WHO AWaRe classification database in the Republic of Kazakhstan as a tool for setting performance targets and guiding optimal use of antibiotics. It will also allow for regular monitoring of antibiotic use and quality surveillance activities, taking into account the most recent evidence on the rational use of antibiotics.

Thus, based on the results of our analysis, we conclude that further research is needed, the results of which will allow the development of a comprehensive policy to contain AMR in Kazakhstan.

The disadvantage of this study is that the data was obtained from free medical care, and does not include data from the private health sector in Kazakhstan, which could provide data on antibiotic over-the-counter dispensing. Unfortunately, the data of private medical institutions on the consumption of antimicrobial drugs, as well as the appointments of doctors from private outpatient clinics are not integrated with the country’s compulsory social and medical insurance system and cannot be officially registered.

## 4. Materials and Methods

This is a retrospective pharmacoepidemiological study conducted using the ATC/DDD (Anatomical Therapeutic Chemical/Defined Daily Dose) methodology, where the calculated average maintenance daily dose of drugs was calculated per 1000 inhabitants per day in Kazakhstan. Monitoring the consumption of antibacterial drugs using the ATC/DDD methodology made it possible to standardize indicators of antibiotic consumption at the international level. This, in turn, allowed us to identify the disadvantages and advantages in the consumption of antibiotics in Kazakhstan, to conduct a comparative analysis.

The calculation of the consumption of antibiotics corrected to the number of inhabitants was carried out according to the formula:

$$\mathrm{DID}=\frac{{\mathrm{DDD}}_{s\;}\times 1000}{\mathrm{population}\;\times \;365\left(\mathrm{or}\;366\right)\;\left(\mathrm{number}\;\mathrm{of}\;\mathrm{days}\;\mathrm{per}\;\mathrm{year}\right)}$$

The DDD values were obtained from the website of the WHO Collaborating Center for Drug Statistics Methodology [24]. It should be noted that this assessment considers only antibiotics with assigned ATC and DDD codes. The exclusion of drugs that do not have DDD values means that the DID estimates obtained in such a way provide an indication of the underestimated consumption of antibiotics in the country.

This paper analyzed the data of the Ministry of Healthcare of the Republic of Kazakhstan on the purchase of medicines by a Single Purchaser and the number of medicines sold for 2017–2019. The Single Purchaser of Kazakhstan is SK Pharmacy, which deals with the centralized procurement of medicines within the guaranteed volume of free medical care in the compulsory social health insurance system. The study did not include data on over-the-counter antimicrobial use.

Data on the population of the country for the study period were taken from the annual statistical compilations “Main socio-economic indicators of the Republic of Kazakhstan” of the Committee on Statistics of the Ministry of National Economy of the Republic of Kazakhstan [25].

The 2019 Access, Watch, and Reserve (AWaRe) classification was used to assess the rational use of antimicrobial agents [4]. According to this classification the Access group includes antibiotics that are active against a wide range of commonly found susceptible pathogens and also show a lower resistance potential than antibiotics from other groups. It includes 48 antibiotics, 19 of which are included in the WHO EML as empirical treatment options of the first or second variants for certain infectious syndromes.

The Watch group includes antibiotics with higher resistance potential and most of the antimicrobial drugs that have a relatively high risk of bacterial resistance selection. Watch group antibiotics should be a priority as key goals of management and monitoring programs. The Watch group includes 110 antibiotics, 11 of which are included in the WHO EML as first or second variants of empirical treatment options for certain infectious syndromes.

The Reserve group includes antibiotics and antibiotic classes that should be used to treat confirmed or suspected infections with multidrug-resistant organisms. A total of 22 antibiotics were assigned to the reserve group. Seven antibiotics of the reserve group are listed in the WHO EML. Traffic light color codes have been proposed to denote different categories: access antibiotics (green), surveillance antibiotics (yellow), and reserve antibiotics (red).

## 5. Conclusions

The results of our study showed a disturbing picture of the irrational consumption of antibiotics of systemic effect in Kazakhstan. There is a negative trend in all three categories of the WHO AWaRe classification. We found that over the period studied, the consumption of Access antibiotics decreased from 39% to 30%. At the same time, as a result of comparing the top 10 most frequently consumed antibiotics, we saw a slightly different picture with fluctuations and a sharp decline in the consumption of Access antibiotics in 2018 (up to 15.5%).

It was revealed that antibiotics of the Watch group are widely consumed in Kazakhstan. In 2019, antibiotics of this group were used in 66.1% of cases of doctor’s prescriptions, which is 4.5% more than in 2017. In 2019, Linezolid entered the top 10 of the most consumed oral antibiotics in the country as a Reserve drug. In general, in Kazakhstan, there is a predominant consumption of parenteral forms of antibiotics of systemic effect. This is probably due not only to the empirical prescription of systemic antibiotics but also to the presence of antibiotic resistance to the drugs of choice. It was also revealed that for the period studied, there was a combined systemic drug in the list of the Single Purchaser, which is included in the WHO list of antibiotics not recommended for use.

Data on the consumption of systemic antibiotics in Kazakhstan show that the current national pharmaceutical policy requires improvement and the inclusion of indicators to improve the rational use of antibiotics and reduce the spread of AMR. There is a need for further research to study the problems of irrational use of antibiotics in more detail and to clarify the reasons for non-compliance with the WHO recommended AWaRe classification.

## Figures and Tables

**Table 1:** The list of antibiotics consumed during the period 2017–2019 according to the WHO AWaRe classification.

№	ATC code	Antibiotic	Class	DID			Category	Listed on EML 2019
				2017	2018	2019		
1	J01AA02	Doxycycline	Tetracyclines	0.067	0.062	0.015	Access	Yes
2	J01BA01	Chloramphenicol	Amphenicols	0.002	0.002	0	Access	Yes
3	J01BA02	Thiamphenicol	Amphenicols	0.001	0.001	0.001	Access	No
4	J01CA01	Ampicillin	Penicillins	0.033	0.041	0	Access	Yes
5	J01CA04	Amoxicillin	Penicillins	0.040	0.008	0	Access	Yes
6	J01CE01	Benzylpenicillin	Penicillins	0.037	0.036	0	Access	Yes
7	J01CR02	Amoxicillin/clavulanic Acid	Beta lactam—beta lactamase inhibitor	0.102	0.110	0	Access	Yes
8	J01DB04	Cefazolin	First-generation cephalosporins	0.501	0.481	0.439	Access	Yes
9	J01DC02	Cefuroxime	Second-generation cephalosporins	0.090	0.084	0.086	Watch	Yes
10	J01DD01	Cefotaxime	Third-generation cephalosporins	0.036	0.034	0.033	Watch	Yes
11	J01DD02	Ceftazidime	Third-generation cephalosporins	0.038	0.037	0.033	Watch	Yes
12	J01DD04	Ceftriaxone	Third-generation cephalosporins	0.363	0.379	0.391	Watch	Yes
13	J01DD08	Cefixime	Third-generation cephalosporins	0.001	0.002	0.001	Watch	Yes
14	J01DD13	Cefpodoxime proxetil	Third-generation cephalosporins	0.001	0.000	0	Watch	No
15	J01DE01	Cefepime	Fourth-generation cephalosporins	0.011	0.009	0.008	Watch	No
16	J01DH02	Meropenem	Carbapenems	0.006	0.007	0.008	Watch	Yes
17	J01DH03	Ertapenem	Carbapenems	0.006	0.004	0.003	Watch	No
18	J01DH04	Doripenem	Carbapenems	0.002	0.002	0.002	Watch	No
19	J01DH51	Imipenem/cilastatin	Carbapenems	0.002	0.001	0.001	Watch	No
20	J01EE01	Sulfamethoxazole/ trimethoprim	Trimethoprim—sulfonamide combinations	0.182	0.010	0	Access	Yes
21	J01FA03	Midecamycin	Macrolides	0.007	0.007	0.005	Watch	No
22	J01FA06	Roxithromycin	Macrolides	0.005	0.004	0.003	Watch	No
23	J01FA09	Clarithromycin	Macrolides	0.165	0.149	0.067	Watch	Yes
24	J01FA10	Azithromycin	Macrolides	0.110	0.157	0.123	Watch	Yes
25	J01GB03	Gentamicin	Aminoglycosides	0.110	0.099	0.084	Access	Yes
26	J01GB04	Kanamycin	Aminoglycosides	0.023	0.018	0.010	Watch	No
27	J01GB06	Amikacin	Aminoglycosides	0.098	0.069	0.055	Access	Yes
28	J01MA01	Ofloxacin	Fluoroquinolones	0.072	0.036	0.031	Watch	No
29	J01MA02	Ciprofloxacin	Fluoroquinolones	0.087	0.079	0.072	Watch	Yes
30	J01MA12	Levofloxacin	Fluoroquinolones	0.753	0.658	0.438	Watch	No
31	J01MA14	Moxifloxacin	Fluoroquinolones	0.061	0.060	0.051	Watch	No
32	J01XA01	Vancomycin (IV)	Glycopeptides	0.006	0.006	0.002	Watch	Yes
33	J01XX01	Fosfomycin (IV)	Phosphonics	0.001	0.001	0.001	Reserve	Yes
34	J01XX08	Linezolid	Oxazolidinones	0	0	0.041	Reserve	Yes

**Table 2:** The list of antibiotics consumed in Kazakhstan during the period 2017–2019, going into EML WHO according to the AWaRe classification.

ATC Code	Antibiotic	Class	Category
J01AA02	Doxycycline	Tetracyclines	Access
J01BA01	Chloramphenicol	Amphenicols	Access
J01CA01	Ampicillin	Penicillins	Access
J01CA04	Amoxicillin	Penicillins	Access
J01CE01	Benzylpenicillin	Penicillins	Access
J01CR02	Amoxicillin/clavulanic Acid	Beta lactam—beta lactamase inhibitor	Access
J01DB04	Cefazolin	First-generation cephalosporins	Access
J01DC02	Cefuroxime	Second-generation cephalosporins	Watch
J01DD01	Cefotaxime	Third-generation cephalosporins	Watch
J01DD02	Ceftazidime	Third-generation cephalosporins	Watch
J01DD04	Ceftriaxone	Third-generation cephalosporins	Watch
J01DD08	Cefixime	Third-generation cephalosporins	Watch
J01DH02	Meropenem	Carbapenems	Watch
J01EE01	Sulfamethoxazole/trimethoprim	Trimethoprim—sulfonamide combinations	Access
J01FA09	Clarithromycin	Macrolides	Watch
J01FA10	Azithromycin	Macrolides	Watch
J01GB03	Gentamicin	Aminoglycosides	Access
J01GB06	Amikacin	Aminoglycosides	Access
J01MA02	Ciprofloxacin	Fluoroquinolones	Watch
J01XA01	Vancomycin (IV)	Glycopeptides	Watch
J01XX01	Fosfomycin (IV)	Phosphonics	Reserve
J01XX08	Linezolid	Oxazolidinones	Reserve

**Table 3:** List of antibiotics consumed in Kazakhstan for the period 2017–2019, not included in the WHO EML according to the AWaRe classification.

ATC Code	Antibiotic	Class	Category
J01BA02	Thiamphenicol	Amphenicols	Access
J01DD13	Cefpodoxime proxetil	Third-generation cephalosporins	Watch
J01DE01	Cefepime	Fourth-generation cephalosporins	Watch
J01DH03	Ertapenem	Carbapenems	Watch
J01DH04	Doripenem	Carbapenems	Watch
J01DH51	Imipenem/cilastatin	Carbapenems	Watch
J01FA03	Midecamycin	Macrolides	Watch
J01FA06	Roxithromycin	Macrolides	Watch
J01GB04	Kanamycin	Aminoglycosides	Watch
J01MA01	Ofloxacin	Fluoroquinolones	Watch
J01MA12	Levofloxacin	Fluoroquinolones	Watch
J01MA14	Moxifloxacin	Fluoroquinolones	Watch

**Table 4:** Share of top 10 antibiotics consumed in Kazakhstan in 2017 according to the WHO classification.

№	Route of Administration	Top 10	DID	Category	Percentage of Use, %	Total Consumption in 2017, DID
1	O	Amoxicillin/clavulanic Acid	0.09	Access	33.0	2.84
2	O	Doxycycline	0.07	Access		
3	O	Amoxicillin	0.04	Access		
4	O	Cefazolin	0.50	Access		
5	P	Gentamicin	0.11	Access		
6	P	Amikacin	0.10	Access		
7	P	Benzylpenicillin	0.04	Access		
8	O	Levofloxacin	0.72	Watch	61.6	
9	O	Clarithromycin	0.16	Watch		
10	O	Azithromycin	0.11	Watch		
11	O	Moxifloxacin	0.06	Watch		
12	O	Ofloxacin	0.05	Watch		
13	O	Ciprofloxacin	0.05	Watch		
14	P	Ceftriaxone	0.36	Watch		
15	P	Cefuroxime	0.07	Watch		
16	P	Ceftazidime	0.04	Watch		
17	P	Levofloxacin	0.04	Watch		
18	P	Cefotaxime	0.04	Watch		
19	P	Ciprofloxacin	0.03	Watch		
20	O	Cefuroxime	0.02	Watch		
J01 Other ABP			0.15	Other	5.4	

**Table 5:** Share of top 10 antibiotics consumed in Kazakhstan in 2018 according to the WHO classification.

№	Route of Administration	Top 10	DID	Category	Percentage of Use, %	Total Consumption in 2018, DID
1	O	Amoxicillin/clavulanic Acid	0.10	Access	15.5	2.65
2	O	Doxycycline	0.06	Access		
3	O	Amoxicillin	0.01	Access		
4	P	Gentamicin	0.10	Access		
5	P	Amikacin	0.07	Access		
6	P	Ampicillin	0.04	Access		
7	P	Benzylpenicillin	0.04	Access		
8	O	Levofloxacin	0.62	Watch	79.2	
9	O	Azithromycin	0.16	Watch		
10	O	Clarithromycin	0.15	Watch		
11	O	Moxifloxacin	0.06	Watch		
12	O	Ciprofloxacin	0.05	Watch		
13	O	Cefuroxime	0.02	Watch		
14	O	Ofloxacin	0.02	Watch		
15	P	Cefazolin	0.48	Watch		
16	P	Ceftriaxone	0.38	Watch		
17	P	Cefuroxime	0.07	Watch		
18	P	Levofloxacin	0.04	Watch		
19	P	Ceftazidime	0.04	Watch		
20	P	Cefotaxime	0.03	Watch		
J01 Other ABP			0.14	Other	5.3	

**Table 6:** Share of top 10 antibiotics consumed in Kazakhstan in 2019 according to the WHO classification.

№	Route of Administration	Top 10	DID	Category	Percentage of Use, %	Total Consumption in 2019, DID
1	O	Doxycycline	0.01	Access	29.6	2.0
2	P	Cefazolin	0.44	Access		
3	P	Gentamicin	0.08	Access		
4	P	Amikacin	0.05	Access		
5	O	Linezolid	0.04	Reserve	2.1	
6	O	Levofloxacin	0.40	Watch	66.1	
7	O	Azithromycin	0.12	Watch		
8	O	Clarithromycin	0.07	Watch		
9	O	Moxifloxacin	0.05	Watch		
10	O	Ciprofloxacin	0.04	Watch		
11	O	Cefuroxime	0.02	Watch		
12	O	Ofloxacin	0.01	Watch		
13	O	Midecamycin	0.01	Watch		
14	P	Ceftriaxone	0.39	Watch		
15	P	Cefuroxime	0.06	Watch		
16	P	Levofloxacin	0.04	Watch		
17	P	Cefotaxime	0.03	Watch		
18	P	Ceftazidime	0.03	Watch		
19	P	Ciprofloxacin	0.03	Watch		
20	P	Ofloxacin	0.02	Watch		
J01 Other ABP			0.04	Other	2.2	

## Data Availability

The data are not publicly available due to the fact that they belong to the Ministry of Health of the Republic of Kazakhstan.

## References

[1] Gulland A. (2017). WHO targets antimicrobial resistance in new essential medicines list. BMJ.

[2] Sharland M., Pulcini C., Harbarth S., Zeng M., Gandra S., Mathur S., Magrini N. (2018). Classifying antibiotics in the WHO Essential Medicines List for optimal use—Be AWaRe. Lancet Infect. Dis..

[3] (2017). World Health Organization Model List of Essential Medicines, 20th list, 2017. Geneva: World Health Organization. https://apps.who.int/iris/handle/10665/273826.

[4] World Health Organization (2019). WHO Model List of Essential Medicines, 21st List.

[5] Zhussupova G., Zhaldybayeva S., Utepova D. (2020). Improving the use of medicines in healthcare organizations to solve the problem of irrational use of medicines in the Republic of Kazakhstan. J. Health Dev..

[6] Analytical Material of the Expanded Board of the Ministry of Health of the Republic of Kazakhstan (2017). Astana: Ministry of Health of the Republic of Kazakhstan.

[7] Budd E., Cramp E., Sharland M., Hand K., Howard P., Wilson P., Wilcox M., Muller-Pebody B., Hopkins S. (2019). Adaptation of the WHO Essential Medicines List for national antibiotic stewardship policy in England: Being AWaRe. J. Antimicrob. Chemother..

[8] Hsia Y., Sharland M., Jackson C., Wong I.C., Magrini N., Bielicki J.A. (2019). Consumption of oral antibiotic formulations for young children according to the WHO Access, Watch, Reserve (AWaRe) antibiotic groups: An analysis of sales data from 70 middle-income and high-income countries. Lancet Infect. Dis..

[9] (2019). WHO Report on Surveillance of Antibiotic Consumption: 2016–2018 Early Implementation, 2019; World Health Organization: Geneva, Switzerland. https://apps.who.int/iris/bitstream/handle/10665/277359/9789241514880-eng.pdf?ua=1.

[10] (2002). WHO Policy Perspectives on Medicines; Promoting Rational use of Medicines: Core Components, 2002; World Health Organization: Geneva, Switzerland. http://archives.who.int/tbs/rational/h3011e.pdf.

[11] Zhussupova G., Skvirskaya G., Reshetnikov V., Dragojevic-Simic V., Rancic N., Utepova D., Jakovljevic M. (2020). The evaluation of antibiotic consumption at the inpatient level in Kazakhstan from 2011 to 2018. Antibiotics.

[12] (2001). WHO Global Strategy for Containment of Antimicrobial Resistance, 2001; World Health Organization: Geneva, Switzerland. https://www.who.int/drugresistance/WHO_Global_Strategy_English.pdf.

[13] Carter B.L. (2016). Evolution of clinical pharmacy in the USA and future directions for patient care. Drugs Aging.

[14] Mahmoud S.H. (2019). Patient Assessment in Clinical Pharmacy: A Comprehensive Guide.

[15] Badreldin H.A., Alosaimy S., Al-jedai A. (2020). Clinical pharmacy practice in Saudi Arabia: Historical evolution and future perspective. J. Am. Coll. Clin. Pharm..

[16] Roman C.P., Dooley M.J., Mitra B. (2019). Emergency medicine pharmacy practice in Australia: A national survey. J. Pharm. Practice Res..

[17] Li J., Li Z. (2018). Differences and similarities in clinical pharmacy practice in China and the United States: A narrative review. Eur. J. Hosp. Pharm..

[18] Abdel-Latif M.M., Sabra K. (2016). Clinical pharmacy practice in Egyptian hospitals. Am. J. Health Syst. Pharm..

[19] Patel H., Gurumurthy P. (2019). Implementation of clinical pharmacy services in an academic oncology practice in India. J. Oncol. Pharm. Pract..

[20] Amiri Jabalbarez F., Dabaghzadeh F., Oghabian Z. (2020). Role of pharmacists in reducing antibiotic prescribing errors in an emergency department. J. Pharm. Pract. Res..

[21] De Rijdt T., Willems L., Simoens S. (2008). Economic effects of clinical pharmacy interventions: A literature review. Am. J. Health Syst. Pharm..

[22] Venkatesh A.N., Keshavamurthy N. (2020). A New Approach to Purchasing of Antibiotics for the Swedish System A Cost-Benefit Analysis of Centralized Purchasing. Master’s Thesis.

[23] Ullman M.A., Parlier G.L., Warren J.B., Mateo N., Harvey C., Sullivan C.J., Bergsbaken R., Mitropoulos I.F., Bosso J.A., Rotschafer J.C. (2013). The economic impact of starting, stopping, and restarting an antibiotic stewardship program: A 14-year experience. Antibiotics.

[24] WHO Collaborating Centre for Drug Statistics Methodology (2019). Guidelines for ATC Classification and DDD Assignment 2020.

[25] Agency for Strategic Planning and Reforms of the Republic of Kazakhstan (2019). Bureau of National Statistics. https://www.gov.kz/memleket/entities/stat?lang=en.

